# The Schistosomiasis SpleenOME: Unveiling the Proteomic Landscape of Splenomegaly Using Label-Free Mass Spectrometry

**DOI:** 10.3389/fimmu.2018.03137

**Published:** 2019-01-22

**Authors:** Miguel Cosenza-Contreras, Renata Alves de Oliveira e Castro, Bruno Mattei, Jonatan Marques Campos, Gustavo Gonçalves Silva, Nívia Carolina Nogueira de Paiva, Rodrigo Dian de Oliveira Aguiar-Soares, Cláudia Martins Carneiro, Luis Carlos Crocco Afonso, William Castro-Borges

**Affiliations:** ^1^Programa de Pós Graduação em Ciências Biológicas, Universidade Federal de Ouro Preto, Ouro Preto, Brazil; ^2^Núcleo de Pesquisas em Ciências Biológicas, Universidade Federal de Ouro Preto, Ouro Preto, Brazil; ^3^Programa de Pós Graduação em Biotecnologia, Universidade Federal de Ouro Preto, Ouro Preto, Brazil; ^4^Departamento de Análises Clínicas, Universidade Federal de Ouro Preto, Ouro Preto, Brazil; ^5^Departamento de Ciências Biológicas, Núcleo de Pesquisas em Ciências Biológicas, Universidade Federal de Ouro Preto, Ouro Preto, Brazil

**Keywords:** *Schistosoma mansoni*, spleen, proteome, host-parasite interactions, helminthiasis, acute inflammation

## Abstract

Schistosomiasis is a neglected parasitic disease that affects millions of people worldwide and is caused by helminth parasites from the genus *Schistosoma*. When caused by *S. mansoni*, it is associated with the development of a hepatosplenic disease caused by an intense immune response to the important antigenic contribution of adult worms and to the presence of eggs trapped in liver tissue. Although the importance of the spleen for the establishment of immune pathology is widely accepted, it has received little attention in terms of the molecular mechanisms operating in response to the infection. Here, we interrogated the spleen proteome using a label-free shotgun approach for the potential discovery of molecular mechanisms associated to the peak of the acute phase of inflammation and the development of splenomegaly in the murine model. Over fifteen hundred proteins were identified in both infected and control individuals and 325 of those proteins were differentially expressed. Two hundred and forty-two proteins were found upregulated in infected individuals while 83 were downregulated. Functional enrichment analyses for differentially expressed proteins showed that most of them were categorized within pathways of innate and adaptive immunity, DNA replication, vesicle transport and catabolic metabolism. There was an important contribution of granulocyte proteins and antigen processing and presentation pathways were augmented, with the increased expression of MHC class II molecules but the negative regulation of cysteine and serine proteases. Several proteins related to RNA processing were upregulated, including splicing factors. We also found indications of metabolic reprogramming in spleen cells with downregulation of proteins related to mitochondrial metabolism. *Ex-vivo* imunophenotyping of spleen cells allowed us to attribute the higher abundance of MHC II detected by mass spectrometry to increased number of macrophages (F4/80^+^/MHC II^+^ cells) in the infected condition. We believe these findings add novel insights for the understanding of the immune mechanisms associated with the establishment of schistosomiasis and the processes of immune modulation implied in the host-parasite interactions.

**Graphical Abstract:**
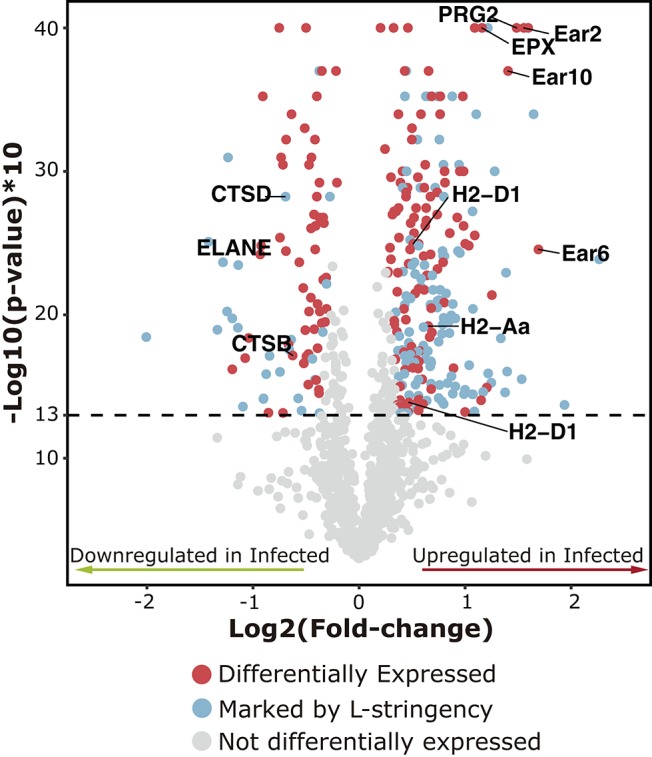
Volcano plot showing the set of proteins used for quantitative analyses in the spleen after 7 weeks of *S. mansoni* infection in the Balb/c mice. Differentially expressed proteins are represented as red dots. Proteins that passed both the fold-change threshold and significance criteria but showed low spectral counts were marked by the L-stringency criteria (Blue dots). Gray dots represent proteins that did not meet the statistical criteria to be considered as differentially expressed.

## Introduction

Schistosomes are long-lived blood-dwelling parasites responsible for causing schistosomiasis, a neglected tropical disease afflicting millions of individuals worldwide (1). Their long persistence in the bloodstream implies they must deploy effective mechanisms to guarantee evasion and modulation of the host immune response (2). A number of proteomic investigations have provided relevant information on the molecular composition of the *S. mansoni* tegument, revealing clues as to how the parasite disguises from the immune system at this host-parasite interface (3–5). Binding of host immunoglobulins and inactivation of complement proteins are proposed strategies but the complex composition and architecture of the tegument offer an unanticipated number of possibilities used by the parasite to circumvent both cellular and humoral responses (6).

Nevertheless, the biology of schistosomes does not guarantee complete masking throughout their residence in the vertebrate host. Once they start feeding on blood, they inevitably regurgitate digestion by-products alongside carried over gut secretions (7). Later, when sexually maturated and paired, female parasites lay a significant number of eggs that ended up trapped in various tissues, in particular the liver (8). There, the eggs containing a viable parasite embryo is capable of protein secretion triggering a granulomatous response around them, ultimately affecting liver homeostasis and function (9). In a previous report we have employed a shotgun proteomic analysis to detect differential expression of liver proteins associated with the onset of oviposition (5 weeks) and at 2 weeks afterwards, when hepatomegaly is fully installed in the murine model of infection (10). In these two time points, we observed a contrasting pattern of protein expression, changing from a “reactive” liver to a “succumbed” tissue due to the intense inflammation induced by parasite antigens. Pioneering observations using 2D-gel based approaches also attested for differential expression of liver proteins during *S. mansoni* infection and possible biomarkers of liver injury found in the serum have been appointed (11, 12).

The spleen, representing another very responsive organ in the context of schistosomiasis, has received little attention in terms of which molecular mechanisms operate once the infection is established. Splenomegaly is a hallmark of the inflammation induced by schistosomes and the understanding of how it reacts to the parasite-derived antigenic burden using both innate and adaptive immune processes could clarify this long lasting host-parasite interplay (13). A great deal of information is now available on the nature of parasitic antigens that are continuously released by adult worms in the circulation (14–16). In this context, both parasite tegument, eggs and alimentary tract are potential sources of a rich molecular arsenal that could ultimately prime and modulate the function of spleen resident cells (17, 18).

To tackle the challenge of deciphering the complex proteome of the responsive spleen, at the peak of acute phase of inflammation, we have chosen a label-free quantitative shotgun strategy based on spectral count as reported by Lundgren et al. (19). Using the spectral count methodology one intends to quantify a protein by the number of MS/MS spectra detected for its encoding peptides. To minimize potential artifacts in protein quantification, a normalized spectral abundance factor (NSAF) has been proposed (20). In this scenario, the total number of spectral counts associated with a given protein is normalized by its length, leading to a good correlation with protein abundance in a sample. While this approach is dependent on high quality MS/MS data, it is worth mentioning that both identification and quantification can be improved simultaneously using optimized and extensive MS/MS spectra collection.

Here we report the identification of 1,565 proteins in the murine spleen proteome after 7 weeks of the *S. mansoni* infection and the differential expression of a set of 325 among them. Upregutaled proteins were mostly representative of major pathways of the adaptive immune response, RNA processing and cell cycle, while most downregulated proteins were associated to catabolic metabolism. Flow cytometry and histology analyses, allowed the establishment of a relationship between the variation of protein abundance measured by proteomics and the exchange in the proportions of subpopulations of spleen cells. The variations in the protein expression within pathways of antigen processing and presentation also suggest the activation of immunomodulatory processes to the presence of parasitic antigens. Thus, the aim of our discovery approach is to shed light into molecular mechanisms governing this successful host-parasite relationship.

## Materials and Methods

### Ethics Statement

All experiments involving animals were conducted in accordance with the Brazilian Federal Legislation (Arouca's Law number 11,794), which regulates the scientific use of animals in Brazil. Both the routine methods for maintenance of the *S. mansoni* life cycle and the experimental procedures involving mice were reviewed and approved by the local Ethics Committee on Animal Experimentation ‘Comissão de Ética no Uso de Animais’ (CEUA), Universidade Federal de Ouro Preto (UFOP), and received the protocol numbers 2017/34 and 2017/35, respectively.

### Infection Model and Experimental Design

The murine model of schistosomiasis was established as follows. Twenty-three female Balb/c mice aged 30 days were anesthetized using a combination of ketamine hydrochloride (8 mg/kg) and xylazine (4 mg/kg) administered via intraperitoneal injection. Continuing, the animals were infected by *S. mansoni* larvae (LE strain) through tail immersion, with a dose of 200 cercariae/animal (Infected group). Control groups were established with 18 non-infected individuals of the same age as those in the Infected group. After 7 weeks of infection, both Infected and Control individuals were weighted and sacrificed using anesthetic overdose. Spleens were weighted to calculate the spleen-to-body ratio as a measure of splenomegaly and then washed and maintained in 2 mL of ice-cold RPMI media for further treatments. Fifteen spleens from infected individuals and thirteen from control were used in the two independent flow cytometry experiments. Eight spleens from Infected individuals and five from Controls were used for histology and mass spectrometry analyses. Tissue sections of approximately 0.5 cm^2^ were collected from these last organs and stored in 3.7% buffered-formalin (pH 7.2) for histological analyses (Supplementary Figure 1).

### Characterization of Schistosome Induced Splenomegaly

To corroborate the establishment of splenomegaly in the mouse model of schistosomiasis, the percentage ratio spleen-to-body weight was calculated as suggested by Henderson et al. (21). Histological analyses were also performed. After fixing in 3.7% buffered-formalin (pH 7.2), spleen sections were embedded in paraffin and tissue sections of approximately 4 μm thick were obtained using a microtome. Then, the slides were stained with hematoxylin & eosin (HE) and Masson's Trichrome, and evaluated for morphological differences between Infected and Control individuals. The presence of *S. mansoni* eggs within stool samples of infected individuals was assessed via the method of spontaneous sedimentation (22), considering the presence of one egg as confirmatory of infection.

### Isolation of Spleen Cells

After extraction, spleens were transferred to a 2 mL bounce glass tissue grinder with 2 mL of RPMI media. The organs were homogenized after 3–4 strokes of the pestle and cells in suspension were then transferred using a Pasteur pipette to a 15 mL tube. Cells were pelleted by centrifugation at 210 g for 10min at 4°C in swing bucket rotor. Splenocytes were then resuspended in 5 mL of lysis buffer (Tris-Base 0.206% w/v, NH_4_Cl 0.77% w/v, pH 7.2) and maintained ice-cold for 2min, in order to promote the lysis of red cells. The lysis was stopped by isotonifying the media after adding 10 mL of PBS (0.1 M, pH 7.2-7.4) and pelleted by centrifugation. Subsequently, isolated spleen cells were washed twice with PBS to eliminate the excess of lysis buffer and stored at −20°C.

### Protein Extraction and Sample Preparation

The isolated spleen cells were resuspended in ammonium bicarbonate buffer (25 mM, pH 8.3), supplemented with 1% protease inhibitor cocktail (PIC, Sigma Aldrich, St. Louis, MO, USA) and then homogenized for 2min by using a tight-fitting Teflon pestle attached to a power drill (Potter S, Braum Biotech International, Melsungen, Germany) set to 1,000 rpm. After homogenization, the samples were transferred to fresh mini-tubes (Axygen, Union City, CA, USA) and stored at −20°C to be used as particulated protein extracts. The protein concentration of each sample was measured on microplates, using the Pierce BCA protein assay kit (Thermo Scientific, Cramlington, UK).

### In Solution Digestion

Three biological replicates were prepared for each group, using 30 μg aliquot from pooled extracts of five animals. Protein extracts were reduced and alkylated before digestion with MS grade trypsin (Promega, Madison, WI, USA) in the presence of the digestion enhancer RapiGest (Waters, UK). Briefly, samples were added with RapiGest to a final concentration of (0.06% w/v) and incubated at 80°C for 10min. Then, proteins were reduced using dithiothreitol (Sigma Aldrich, St. Louis, MO, USA) to a final concentration of 3.3 mM at 60°C for 10min. Alkylation was achieved using iodoacetamide (GE Healthcare, Little Chalfont, Buckinghamshire, UK) to a final concentration of 9.4 mM at room temperature in the dark, for 30min. Samples were digested using sequencing grade trypsin at a ratio of 50:1 (protein/trypsin) and incubated at 37°C for 16 h. To stop the digestion and precipitate RapiGest, trifluoroacetic acid was added to a final concentration of 0.5% v/v, resulting in a pH ≤ 2. After that, a centrifugation step was performed at 20,000 g for 15min at 7°C (Mikro 200R, Hettich Zentrifugen, Tuttlingen, DEU) for the removal of insoluble particles.

### Liquid Chromatography–Mass Spectrometry

After digestion, 0.5 μg of peptide samples solubilized in 2% acetonitrile-0.1% trifluoroacetic acid solution (TFA, Sigma Aldrich, St. Louis, MO, USA) were injected into the nanoUHPLC UltiMate® 3,000 (Dionex, San Jose, USA) system. In first instance, peptides were trapped on a Nano-Trap Acclaim PepMap100 C18 column (100 μm i.d. × 2 cm, 5 μm, 100 Å; Thermo Scientific, Waltham, MA, USA) and washed for 3min in 2% acetonitrile (ACN, HPLC grade, USE)/water/0.1% TFA solution, at a flow rate of 7 μL/min. After that, a reverse phase chromatography was performed using an Acclaim PepMap18 RSLC (75 μm i.d. × 15 cm, 2 μm, 100 Å; Thermo Scientific) column (in tandem with the previously mentioned column), maintained at 40°C with a constant flow rate of 0.3 μL/min. Following, a multistep gradient of solvents A (0.1% Formic Acid, HPLC grade, JTBaker, Mexico) and B (80% ACN, 0.1% Formic Acid) was executed as follows: a conditioning step with 3.8% of B was applied on the first 3min, followed by a ramp from 3.8 to 30% B over 120min and 30–50% B between 120 and 150min, with a final ramp with 99% B to 162min and a reconditioning step with 3.8% B until 180min.

The spectral data was acquired using a Q Exactive mass spectrometer (Thermo Scientific®, Bremen, Germany) operating at full-scan/MS2 mode. The ion source was set at 3.8 kV with a capillary temperature of 250°C. The acquisition method for the survey scans was tuned for a resolution of 70,000 at *m/z* 200, with a mass range between 300 and 2,000 *m/z*, and a AGC target of 1e^6^ ions in up to 120 ms. For MS/MS acquisitions, a Top 12 criteria was used, isolating up to 12 most intense precursors ions with charge state between +2 and +4 from a 1.2 *m/z* window, excluding isotopes. Selected ions were fragmented by Higher Energy Collision Induced Dissociation (HCD) with a stepped Normalized Collisional Energy (NCE) of 28–30. After collision, the product ion spectra were acquired with a resolution of 17,500 with a maximum injection time of 60 ms and a target value of 5e^5^ (minimum AGC target 6.25e^3^) and a Dynamic exclusion time of 40 s.

### Analysis of Proteomic Data

After gathering the spectral data, it was submitted to database search for protein identification and quantification using Patternlab for protemics software (version 4.1.0.6) (23). The Uniprot sequence database from *Mus musculus* (59,517 total sequences) was downloaded and later processed to include also reverse sequences of each protein (labeled as decoy) resulting in a mixed target-decoy database (eliminating subset sequences) which was used as background for the identification algorithm. For spectrum match search, the Comet algorithm was used to compare the experimental tandem mass spectra against those theoretical spectra generated from target and decoy sequences. The parameters applied to establish the theoretical spectra were generated from an *in silico* digestion of the protein database, which was set to mimic a semi-specific tryptic digestion with up to 2 missed cleavage sites, including cysteine carbamidomethylation (+57.02146 Da) as fixed and methionine oxidation (+15.9949 Da) as variable post-translational modifications (up to 3 variable modifications for peptide), a mass range MS1 precursor peptide of 550–5,500 Da with a mass tolerance error of 10 ppm and considering MS2 ions from B, Y and neutral loss fragmentation. This resulted in a list of candidates for each spectrum, which was later filtered using the Search Engine Processor (SEPro). In this step, pre-processing quality filters were applied as follows: delta mass of 30 ppm, a total DeltaCN of 0.001 and at least 6 amino acids on peptide sequence and post-processing quality filters applying to accept only proteins that have at least 1 peptide, 1 spectral count, PSM with a delta mass of 10 ppm from the average delta mass obtained and a Normalized primary score (XCorr) of at least 2.0 for proteins identified with only 1 mass spectrum and at least 1.8 for proteins identified with at least 2 mass spectra. Identifications were grouped by number of enzymatic termini and a Bayesian score, which included XCorr, delta CN, peaks matched values, delta mass error, spectral count score and secondary rank, was used to sort the identification in a non-decreasing order of confidence. Later, a cutoff score was applied to accept a false-discovery rate (FDR) of 3% for spectra, 2% for peptide and 1% for protein identification based on the number of labeled decoys into the identification list.

Quantification was based on the number of peptide spectral counts, normalized using the spectral abundance factor (NSAF) (20). The relative quantification of proteins was performed using the TFold method (24), setting the F-stringency to 0.03 and the L-stringency to 0.4. The BH q-value threshold for the significance test was set to 0.05. These parameters were set to reach a fold-change threshold of ~1.2 and a *p*-value of 0.05, as the criteria to consider proteins as differentially expressed. The whole quantitative data, along with the set of differentially expressed proteins, was exported into a CSV file for enrichment analysis and data visualization using the R programming language (version 3.5.1) running on R Studio (version 1.1.442). The reproducibility of the protein quantifications was evaluated through the Pearson's correlation test between each biological replicate (Supplementary Figure 2).

Enrichment analysis based on Reactome categories was performed using the package “ReactomePA” (25) and the package “clusterProfiler” (26) was used for the categorization based on KEGG pathways. The hypergeometric model was used as the statistical method for enrichment and categorization. All categories with a geneset size between 100 and 1,000 were tested, with a significance threshold per enriched category set to *P* ≤ 0.05 with FDR adjustment. For the visualization of the functional enrichment results, both the “enrichplot” (27) and “pathview” (28) packages were used.

### *Ex-vivo* Immunophenotyping of Splenocytes

Fifteen infected mice and 13 controls were used for immunophenotyping via flow cytometry experiments. After isolation (see Isolation of Spleen Cells), spleen cells were counted and resuspended at a concentration of 1 × 10^7^/mL. Then, a total of 5×10^5^ splenocytes were transferred to fresh polystyrene tubes in the presence of 0.5 μg IgG anti-Fc receptors CD32 and CD16 and incubated for 5min at room temperature. Cells were then incubated with the diluted anti-mouse cell surface monoclonal antibody (mAb) for 30min, at room temperature (RT), in the dark. The following mAbs were used: 0.25 μg of anti-CD3ε (PE, clone 145-2C11, BioLegend); 0.025 μg of anti-CD4 (FITC, clone FM4-5, BioLegend); 0.125 μg of anti-CD8 (PE-Cy7, clone 53-6.7, BD Pharmigen); 0.1 μg of anti-F4/80 (PE, clone BM8, BioLegend); 0.03 μg of anti-MHC II (Alexa Fluor 647, clone M5/114.15.2) and 0.5 μg of anti-CD11c (PE-Cy7, clone HL3, BD Pharmingen). The splenocytes were then washed in PBS pH 7.2 and centrifuged at 400 *g* for 10min at RT. Then, the labeled cells were fixed for 30min at RT, with 200 μL of FACS fix solution (10 g/L paraformaldehyde, 1% sodium cacodylate, 6.67 g/L sodium chloride, pH 7.2). The staining was performed in two different tubes with the respective panels of mAbs: (1) CD3-PE, CD4-FITC and CD8-PECy7–to measure the frequencies of subpopulations of T lymphocytes and (2) F4/80-PE, CD11c-PECy7 and MHC-II-AF647–to analyze the subpopulations of antigen-presenting cells (macrophages and dendritic cells) expressing MHC-II. The data were collected using a flow cytometer FACSCalibur (Becton Dickinson, USA) and a total of 50,000 events were acquired for each tube. The Cell-Quest software package was used for data acquisition. Data analysis was performed using the FlowJo software (Tree Star, USA). Gating strategies were used according to the subpopulation of splenocytes studied and were representative of the whole population of spleen cells (Supplementary Figure 3A) and depicted by the pseudocolor bi-dimensional fluorescence graphs. For T-lymphocytes, two cell scatter plots were used by evaluating the FL2/anti-CD3 PE vs. FL1/anti-CD4 FITC and FL2/anti-CD3 PE vs. FL3/anti-CD8 PE-Cy7. The gating strategy involved identification of the double-positive quadrant representative of the CD3^+^/CD4^+^ and CD3^+^/CD8^+^ T-lymphocyte subpopulations (Supplementary Figure 3B). Antigen-presenting cells (APCs) were identified based on the expression of the membrane marker CD11c (dendritic cells) using the FL3/anti-CD11c vs. SSC (Supplementary Figure 3C) and the marker F4/80 (macrophages) using the FL2/anti-F4/80 vs. SSC (Supplementary Figure 3D). The proportion of each APC expressing MHC-II was identified based on the correspondent parent gate as illustrated in the Supplementary Figures 3C and 3D, and these proportions were extrapolated to the whole population of spleen cells (percentage of grandparent). The results were expressed as percentage of positive cells within the whole population (Supplementary Table 1). The percentages of cell populations for each sample were exported into Excel files and loaded into R for data analysis. The Shapiro-Wilk normality test was performed for each cell population under investigation. To evaluate the differences between Infected and Control individuals, the Student's *t*-Test was performed in cases of parametric data and the Wilcoxon test in cases of non-parametric.

## Results

### Characterization of the Splenomegaly in the Murine Model

**Figure 1:**
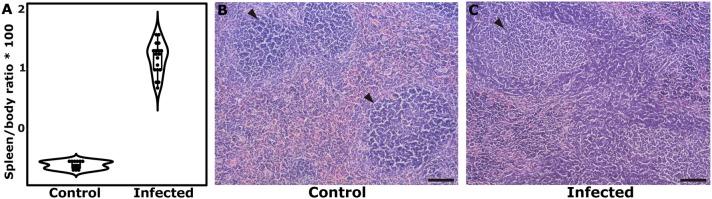
Corroboration of the establishment of splenic disease after 7 weeks of *S. mansoni* infection. **(A)** Dot and box plots showing the differences in the median Spleen/body ratio and the interquartile range. **(B)** Histological aspect in the spleen of a control individual, showing the normal organization of the white and red pulps, with primary follicle predominance (arrow). **(C)** Histological aspect in the spleen of an infected mouse, showing large lymphatic follicles with prominent germinal centers. Reference bar represents 100 μm.

In first instance, the spleen-to-body weight ratio was used as a measure of splenomegaly as previously suggested (21). The spleen-to-body ratio (gram/gram × 100) was significantly augmented in infected individuals (2.24 ± 0.184) when compared to uninfected controls (0.475 ± 0.0149) (*P*-value of unpaired Student's *t*-test < 0.001) (Figure 1A). The parasitological examination allowed confirmation of an active infection through the detection of eggs in the feces of infected mice. The histological analyses of the spleen also attested for morphological differences between infected and control individuals. In non-infected controls, the splenic parenchyma showed normal histology. The white pulp is formed by the periarteriolar sheaths of lymphocytes with lateral expansions forming lymphatic follicles frequently without germinal centers (primary follicles) (Figure 1B). In infected animals there is a significant expansion of the white pulp, markedly of the lymphatic follicles that present with evident germinal centers and expansion of mantle and marginal zones (secondary follicles) (Figure 1C) and an increased number of lymphocytes and phagocytes in the region of the red pulp are observed. It was not possible to delimitate the expansion of the lymphatic follicles in the spleens of infected individuals due to the intense hyperplasia. With these observations, along with the positivity in the copro-parasitological test, we were able to corroborate the establishment of the splenomegaly associated with the acute phase of inflammation in the murine model (21).

### The Composition of the Spleen Proteome

We were able to identify 3,326 total proteins resulting in 1,565 grouped entries on maximum parsimony from both infected and uninfected individuals (see data availability statement). The FDR for these identifications was reported to be 0.05% for spectra, 0.08% for peptides and 0.10% for proteins. We then used the normalized spectral abundance factor (NSAF) as the measure of abundance (20).

**Figure 2:**
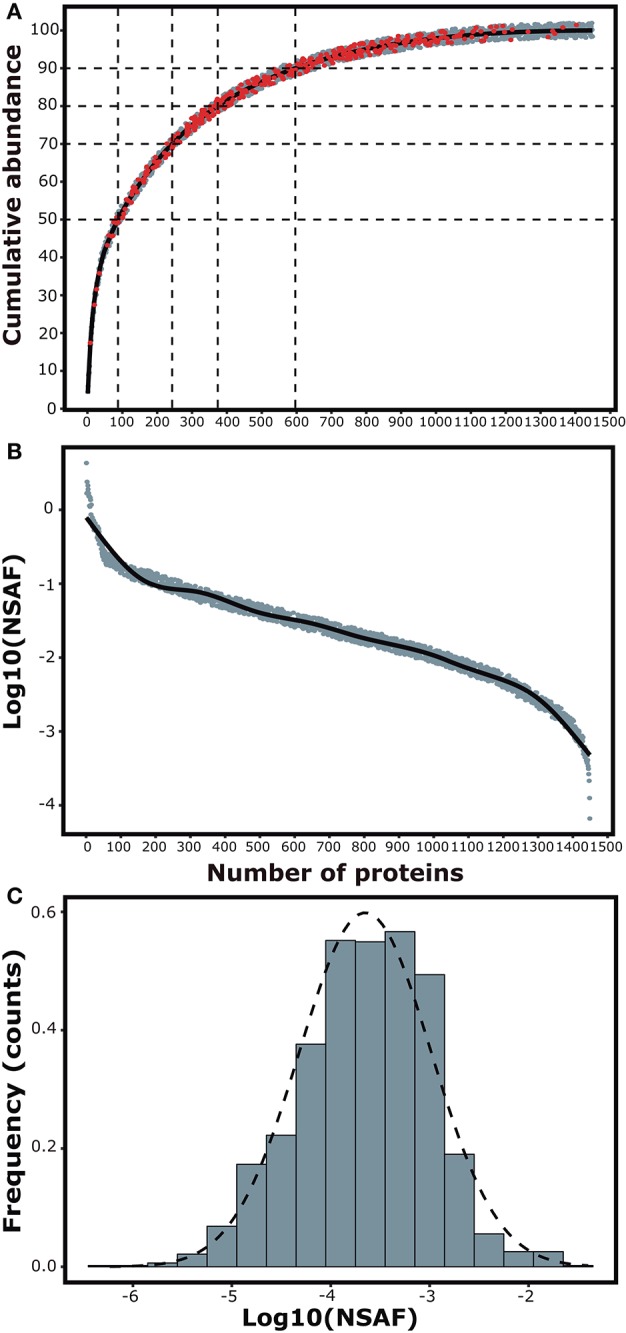
Composition analyses of the spleen proteome. **(A)** Cumulative abundance plot shows the total number of identified proteins in ascending order in relation to their mass contribution to the spleen proteome. Red dots represent differentially expressed proteins. **(B)** Dynamic abundance plot showing the variability in abundance of the identified proteins. **(C)** Histogram for the frequency distribution of protein abundance in the samples.

The cumulative abundance plot shows the arrangement of the proteins in ascending order as a representation of the contribution of each protein to the total mass of the spleen proteome. Notably, 50% of the total protein extract is accounted by the abundance of 88 proteins, mostly represented by structural proteins (Filamin, Lamin, Actin, Tubulin, Talin-1, Cofilin, Coronin, among others), histones and some associated with mitochondrial metabolism. Only 10% of the extract could be represented by over 720 lowly abundant proteins (Figure 2A). To assess the dynamic range of protein abundance, we plotted the log10 of the spectral counts for each of the proteins arranged according to their contribution to the total mass. It was observed a variation of more than 4 orders of magnitude between the most and the less abundant protein in the samples (Figure 2B). A histogram for the logarithm of the spectral counts was used to evaluate the frequency distribution of protein abundance in spleen cells. The graph appears visually skewed toward more abundant components (Figure 2C). This observation was tested via the Shapiro-Wilk normality test, proving a non-Gaussian distribution (*P*-value = 0.0006307).

### The Differential Expression of Proteins in the Spleen at 7 Weeks Post Infection

The TFold test underpinned by the Patternlab software yielded NSAF values of the identified proteins to detect the differential expression of 325 proteins in the spleen of infected individuals. Among these proteins, 242 were found upregulated in the experimental condition and 83 were downregulated. One hundred and fifty proteins were marked by the L-Stringency filter, indicating that they passed the fold-change and significance criteria but exist in low abundance in the samples. In practical terms, we found that 20.7% of the detected proteins in the spleen are differentially expressed, and 74.5% of them are found upregulated (Graphical Abstract). The whole set of proteins used for the quantitative analysis is available in the Supplementary Table 2.

### Functional Enrichment of Cellular Pathways Shows the Development of an Adaptive Immune Response

The differentially expressed proteins were used to feed a hypergeometric model for their functional categorization based on the Reactome database. Among these proteins, 218 had mappings within Reactome categories and were tested in the model. After the analysis, 55 pathways were significantly enriched (*P*-value < 0.05, controlled by FDR). We manually dropped enriched categories which were considered to have too general or specific pathways with redundant proteins, ending up with 27 functional terms. A heatplot was employed in order to visualize the quantitative information of the proteins enriching each pathway and to determine if particular pathways were better represented by upregulated or downregulated proteins.

In general terms, the differentially expressed proteins were categorized within pathways associated with innate and adaptive immunity (Neutrophil degranulation, Antigen processing and presentation, TCR signaling and Signaling by interleukins), RNA processing (Translation and Splicing), Cell cycle (Synthesis of DNA, replication and packaging), vesicle transport, and catabolic metabolism (TCA cycle and electron transport, Metabolism of amino acids).

#### Pathways Associated With the Immune Response Are Mostly Enriched by Upregulated Proteins

**Figure 3:**
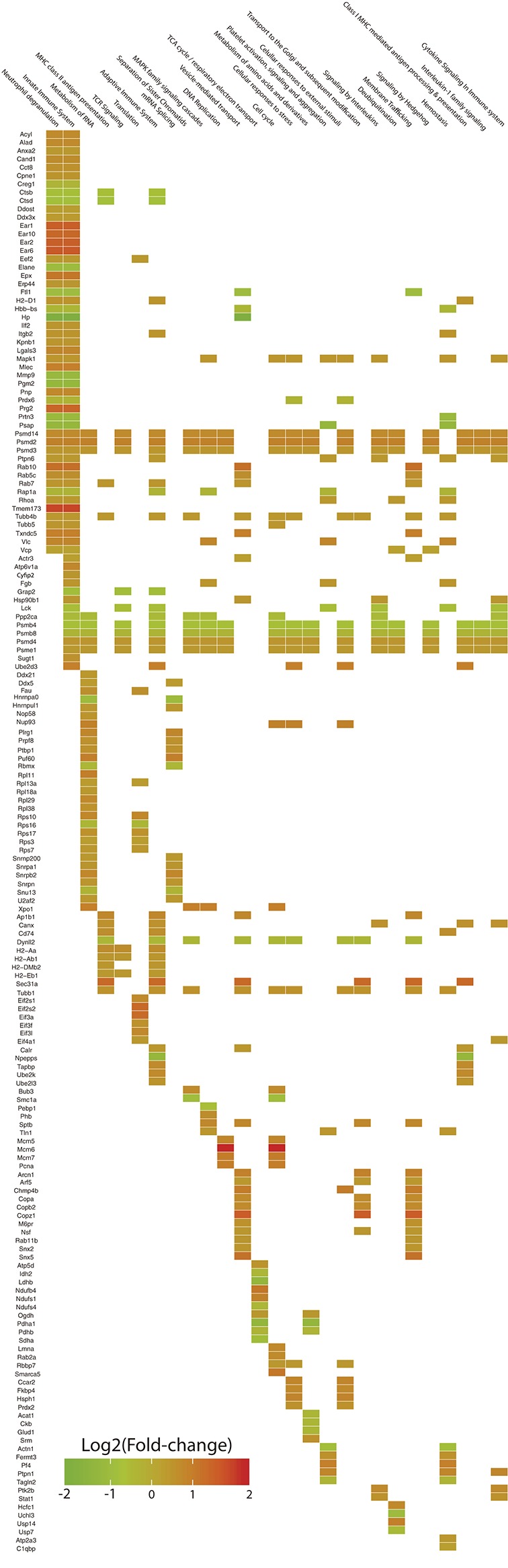
Heatplot for the visualization of the functional enrichment analysis based on *Reactome* categories. The expression levels of 218 differentially expressed proteins are showed in the vertical axis in association with 27 cellular pathways in which they are included (horizontal axis). Squares in the intersection between a particular protein and a category, indicate the presence of the protein within the pathway. The color of the square represents the expression level of the protein as the Log2 of the fold-change.

The pathways with better representation were those related to the immune response. Major pathways such as Innate immune system and Neutrophil degranulation included more than 25% of the mapped proteins and these were mostly upregulated (>70%). Within the aforementioned categories, we observed a pronounced enrichment for eosinophil granule proteins such as eosinophil-associated ribonucleases (Ear1, Ear2, Ear6, and Ear10; Fold-change: +3.02, +2.94, +3.26, +2.65, respectively) and eosinophil peroxidase (Epx; Fold-change: +2.24), along with bone marrow proteoglycan 2 (Prg2; Fold-change: +2.80), galectin-3 (Lgals3; Fold-change: +1.74), malectin (Mlec; Fold-change: +1.99), purine-nucleoside phosphorylase (Pnp; Fold-change: +1.68) and the SGT1 protein homolog (Sugt1; Fold-change: +1.52). It was also found upregulation of STAT1 transcription factor (Stat1; Fold-change: +1.47) and protein-tyrosine kinase 2-beta (Ptk2b; Fold-change: +1.60), both of them categorized within the signaling pathway of interleukins. On the other hand, haptoglobin (Hp; Fold-change: −4.02), hemoglobin (Hbb-bs; Fold-change: −1.34), matrix metallopeptidase 9 (Mmp9; Fold-change: −2.14), proteinase-3 (Prtn3; Fold-change: −2.06) and neutrophil elastase (Elane; Fold-change: −1.90) were found downregulated (Table 1; Figure 3).

**Table 1:** Consolidated list of differentially expressed proteins after 7 weeks of *S. mansoni* infection in female Balb/c mice.

**UniProtKB Entry**	**Protein name (Gene code)**	**Fold-change (NSAF ratio) Infected/Control**	***P*-value**	**L-Stringency marked**
P97426	Eosinophil-associated, ribonuclease A family, member 1 (Ear1)	+3.02	<0.0001	No
W0UVF7	Eosinophil-associated, ribonuclease A family, member 2 (Ear2)	+2.94	<0.0001	No
Q923L7	Eosinophil-associated, ribonuclease A family, member 6 (Ear6)	+3.26	0.0034	No
Q923L6	Eosinophil-associated, ribonuclease A family, member 10 (Ear10)	+2.65	0.0001	No
P49290	Eosinophil peroxidase (Epx)	+2.23	<0.0001	No
Q545D8	Bone marrow proteoglycan 2 (Prg2)	+2.80	<0.0001	No
P16110	Galectin-3 (Lgals3)	+1.74	0.0081	No
Q6ZQI3	Malectin (Mlec)	+1.99	0.0333	Yes
P23492	Purine-nucleoside phosphorylase (Pnp)	+1.68	0.0153	Yes
Q9CX34	SGT1 protein homolog (Sugt1)	+1.52	0.0163	Yes
Q61646	Haptoglobin (Hp)	−4.02	0.0142	Yes
A8DUK4	Hemoglobin (Hbb-bs)	−1.34	0.0019	No
P41245	Metallopeptidase 9 (Mmp9)	−2.14	0.0436	Yes
A0A0R4IZY6	Proteinase 3 (Prtn3)	−2.06	0.0144	No
Q3UP87	Neutrophil elastase (Elane)	−1.90	0.0032	No
P01897	H-2 class I histocompatibility antigen, L-D alpha chain (H2-D1)	+1.32	0.0437	No
P14427	H-2 class I histocompatibility antigen, D-P alpha chain (H2-D1)	+1.38	0.0407	No
P01900	H-2 class I histocompatibility antigen, D-D alpha chain (H2-D1)	+1.42	0.0031	No
P68037	Calnexin (Canx)	+1.51	0.0281	No
P14211	Calreticulin (Calr)	+1.29	0.0028	No
A0A0G2JGL0	Ubiquitin-conjugating enzyme E2 D3 (Ube2d3)	+1.99	0.0475	No
Q5HZK3	Proteasome activator subunit 1 (PA28) (Psme1)	+1.28	0.013	No
P14685	Proteasome 26S subunit, non-ATPase 3 (Psmd3)	+1.40	0.0029	Yes
O35593	Proteasome 26S subunit, non-ATPase 14 (Psmd14)	+1.60	0.0301	Yes
P99026	Proteasome subunit, beta type 4 (Psmb4)	−1.55	0.0003	No
G3UZW8	Proteasome subunit, beta type 8 (Psmb8)	−1.27	0.0131	Yes
P04228	H-2 class II histocompatibility antigen, A-D alpha chain (H2-Aa)	+1.57	0.0119	No
Q31099	H-2-M beta 2 (class II histocompatibiity antigen) (H2-DMb2)	+1.41	0.0044	Yes
P01921	H-2 class II histocompatibility antigen, A-D beta chain (H2-Ab1)	+1.34	0.0181	No
P01915	H-2 class II histocompatibility antigen, E-D beta chain (H2-Eb1)	+1.29	0.0003	No
P04441	CD74 antigen (Cd74)	+1.56	0.0052	No
A2AQ07	Tubulin beta-1 chain (Tubb1)	+1.47	0.0031	No
P68372	Tubulin beta-4B chain (Tubb4b)	+1.33	0.0009	No
Q9QVP9	Protein-tyrosine kinase 2-beta (Ptk2b)	+1.60	0.021	Yes
A0A087WSP5	Signal transducer and activator of transcription 1 (Stat1)	+1.47	0.001	No
Q3UPL0	Sec31 homolog A (Sec31a)	+2.63	0.0248	Yes
P10605	Cathepsin B (Ctsb)	−1.54	0.0191	No
Q3UCD9	Cathepsin D (Ctsd)	−1.61	0.0014	Yes
Q9D0M5	Dynein light chain LC8-type (Dynll2)	−1.33	0.0034	No
Q9JIK5	Nucleolar RNA helicase 2 (Ddx21)	+1.31	0.0453	No
A0A087WQ46	Nucleolar protein 58 (Nop581)	+1.44	0.0213	Yes
Q6P5F9	Exportin-1 (Xpo1)	+1.98	0.0356	Yes
Q6P4T2	U5 small nuclear ribonucleoprotein 200 (Snrnp200)	+1.30	0.0269	Yes
P63163	Small nuclear ribonucleoprotein N(Snrpn)	+1.41	0.0166	No
P57784	U2 small nuclear ribonucleoprotein polypeptide A (Snrpa1)	+1.38	0.0473	No
P26369	Splicing factor U2AF (U2af2)	+1.31	0.0486	Yes
Q9CQI7	U2 small nuclear ribonucleoprotein B(Snrpb2)	+1.75	0.0009	No
Q8VDM6	Heterogeneous nuclear ribonucleoprotein U-like 1 (Hnrnpul1)	+1.30	0.014	Yes
B7ZC27	Pre-mRNA processing factor 8 (Prpf8)	+2.25	0.0345	Yes
Q3UEB3	Poly-U binding splicing factor 60 (Puf60)	+1.93	0.0036	Yes
D3Z4V1	Pleiotropic regulator 1(Plrg1)	+1.74	0.0038	Yes
F7AXP1	Polypyrimidine tract binding protein 1 (Ptbp1)	+1.48	0.0389	No
Q9CX86	Heterogeneous nuclear ribonucleoprotein A0 (Hnrnpa0)	−1.91	0.0037	No
P63330	Protein phosphatase 2 (Ppp2ca)	−1.41	0.0194	No
Q9WV02	RNA binding motif protein (Rbmx)	−1.33	0.0023	No
Q9D0T1	Non-histone chromosome protein (Nhp2l1)	−1.28	0.0001	No
Q5M9M0	Ribosomal protein L13A (Rpl13a)	+1.24	0.0019	No
Q5M9K7	Ribosomal protein S10 (Rps10)	+1.75	0.0011	No
P14131	Ribosomal protein S16 (Rps16)	−1.32	0.0002	No
Q5M9L7	Ribosomal protein S17 (Rps17)	+1.48	0.0211	No
Q5YLW3	Ribosomal protein S3 (Rps3)	+1.23	0.001	No
P62082	Ribosomal protein S7 (Rps7)	+1.30	0.0392	No
P58252	Eukaryotic translation elongation factor 2 (Eef2)	+1.28	0.0068	No
Q6ZWX6	Eukaryotic translation initiation factor 2, subunit 1 alpha (Eif2s1)	+1.58	0.0037	No
Q3ULL5	Eukaryotic translation initiation factor 2, subunit 2 (beta) (Eif2s2)	+2.61	0.005	Yes
P23116	Eukaryotic translation initiation factor 3, subunit A (Eif3a)	+2.53	0.0145	Yes
Q9DCH4	Eukaryotic translation initiation factor 3, subunit F (Eif3f)	+1.46	0.0206	No
Q8QZY1	Eukaryotic translation initiation factor 3, subunit L (Eif3l)	+1.67	0.0079	Yes
Q5F2A7	Eukaryotic translation initiation factor 4A1 (Eif4a1)	+1.29	0.0017	No
P61027	RAB10, member RAS oncogene family (Rab10)	+2.38	0.0072	No
P46638	RAB11B, member RAS oncogene family (Rab11b)	+1.38	0.0043	No
Q8C266	RAB5C, member RAS oncogene family (Rab5c)	+1.55	0.0021	No
P51150	RAB7, member RAS oncogene family (Rab7)	+1.40	0.0185	No
F8WHL2	Coatomer protein complex subunit alpha (Copa)	+1.60	0.0215	Yes
O55029	Coatomer protein complex, subunit beta 2 (beta prime) (Copb2)	+1.62	0.0066	Yes
P61924	Coatomer protein complex, subunit zeta 1 (Copz1)	+3.13	0.0003	Yes
Q5XJY5	Archain 1 (Arcn1)	+1.88	0.0105	Yes
Q9CWK8	Sorting nexin 2 (Snx2)	+1.36	0.0001	Yes
Q9D8U8	Sorting nexin 5 (Snx5)	+2.32	<0.0001	Yes
Q9D8B3	Charged multivesicular body protein 4B (Chmp4b)	+2.10	0.0358	Yes
Q3UGX2	Spectrin beta (Sptb)	+1.73	0.0116	Yes
Q3UKQ5	Mannose-6-phosphate receptor (M6pr)	+1.47	0.0236	No
Q3UFJ3	Pyruvate dehydrogenase E1 alpha 1 (Pdha1)	−2.36	0.0007	Yes
P16125	Lactate dehydrogenase B (Ldhb)	−2.29	0.0105	Yes
Q8K2B3	Succinate dehydrogenase complex, subunit A, flavoprotein (Fp) (Sdha)	−1.68	0.0028	No
Q9CXZ1	NADH dehydrogenase (ubiquinone) Fe-S protein 4 (Ndufs4)	−1.32	0.0284	No
P54071	Isocitrate dehydrogenase 2 (NADP+), mitochondrial (Idh2)	−1.31	0.0014	No
Q9D051	Pyruvate dehydrogenase (lipoamide) beta (Pdhb)	−1.30	0.0001	Yes
Q04447	Creatine kinase (Ckb)	−1.33	0.0283	Yes
Q91VD9	NADH dehydrogenase (ubiquinone) Fe-S protein 1 (Ndufs1)	+1.85	0.0083	Yes
Q9CQC7	NADH dehydrogenase (ubiquinone) 1 beta subcomplex 4 (Ndufb4)	+2.22	0.0393	No
Q9D3D9	ATP synthase, mitochondrial F1 complex, delta subunit (Atp5d)	+1.45	0.0305	Yes
P97311	DNA replication licensing factor (Mcm6)	+4.80	0.004	Yes
Q91ZW3	SWI/SNF-related actin-dependent regulator of chromatin (Smarca5)	+2.15	0.0003	Yes
Q52KC3	DNA helicase (Mcm5)	+1.74	0.0104	Yes
Q3U4T8	DNA helicase (Mcm7)	+2.10	0.0018	Yes
Q542J9	Proliferating cell nuclear antigen (Pcna)	+2.05	0.0032	No
Q8BJ71	Nucleoporin 03 (Nup93)	+1.83	0.0267	Yes
P48678	Lamin A (Lmna)	+1.70	0.0002	No
Q9WVA3	Mitotic checkpoint protein BUB3 (Bub3)	+1.60	0.0089	No
Q60973	Histone binding protein 7 (Rbbp7)	+1.31	0.0315	No
Q9CU62	Structural maintenance of chromosomes protein 1A (Smc1a)	−1.79	0.0192	Yes
Q4FK28	Adenosine deaminase (Ada)	+1.47	0.018	Yes

#### Major Components of Antigen Processing and Presentation Pathways Were Found Differentially Expressed

Representative pathways of the Adaptive Immune System and other associated subcategories were significantly enriched, including 17% of the mapped proteins with 66% of them upregulated. Both MHC class I and II antigen presenting pathways were significantly enriched in this analysis with both upregulated and downregulated proteins. In terms of MHC class I antigen processing and presentation, upregulation of three Class I histocompatibility antigens (H2-D1 L-D alpha chain, D-P alpha chain and D-D alpha chain; Fold-change: +1.32, +1.38, +1.42, respectively) was observed in parallel to an increased expression of calnexin and calreticulin cheperones (Canx, Calr; Fold-change: +1.51, +1.29, respectively). Also one activator subunit and two regulatory subunits of the proteasome were found upregulated (Psme1, Psmd3, and Psmd14; Fold-change: +1.28, +1.40, +1.60, respectively), along with the ubiquitin-conjugating enzyme E2D 3 (Ube2d3; Fold-change: +2.00). In contrast, two proteolytic subunits of the proteasome (Psmb4, Psmb8; Fold-change: −1.55, −1.27, respectively) and the aminopeptidase puromycin sensitive protein (Npepps; Fold-change: −2.21) were found downregulated in the infective condition (Table 1; Figure 3).

**Figure 4:**
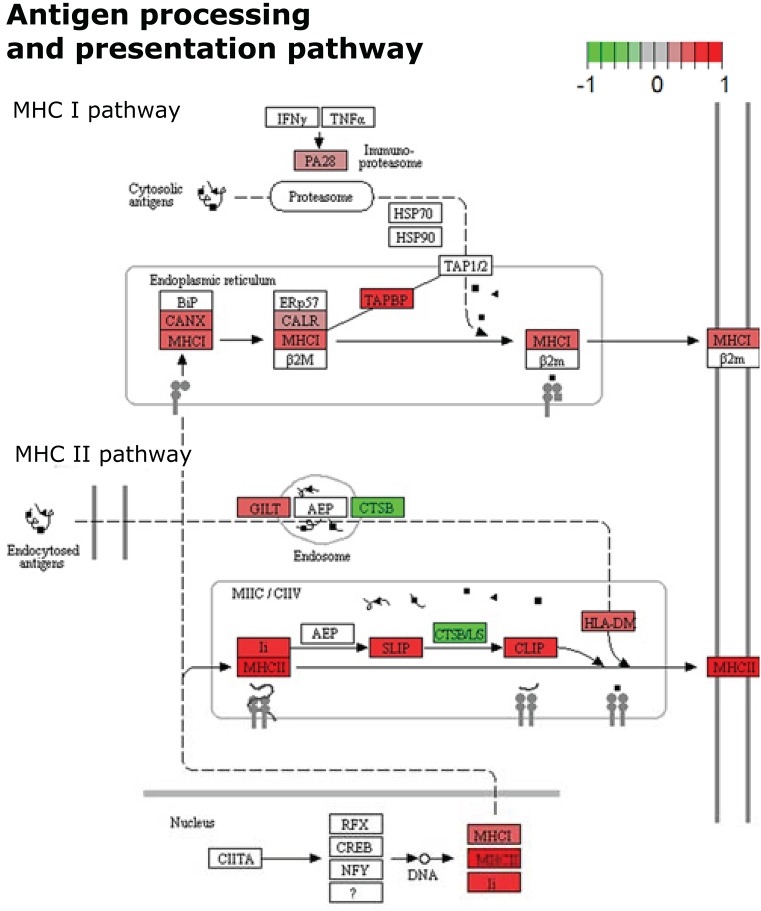
KEGG Pathway enrichment result visualization from differentially expressed proteins in spleen cells after 7 weeks of *S. mansoni* infection. Color marks represent the expression levels of proteins participating in the cellular pathway of Antigen processing and presentation. Uncolored proteins were neither identified nor differentially expressed after quantitative analyses.

Within the MHC class II antigen presentation pathway, we found the upregulation of four histocompatibility antigens (H2-Aa, H2-DMb2, H2-Ab1, H2-Eb1; Fold-change: +1.57, +1.41, +1.34, +1.29, respectively), the CD74 antigen (Cd74, Fold-change: +1.56) and traffic-related proteins such as tubulins (Tubb1, Tubb4b; Fold-change: +1.47, +1.33, respectively), the Sec31 homolog (Sec31a; Fold-change: +2.63) and RAB7 (Rab7, Fold-change: +1.41). We also observed the downregulation of two cathepsins (Ctsd, Ctsb; Fold-change: −1.62, −1.54, respectively) and the dynein light chain LC8-type (Dynll2; Fold-change: −1.33). It is worth mentioning that the NSAF for MHC class II histocompatibility antigens is one order of magnitude higher when compared to MHC class I histocompatibility antigens, indicating a higher relative expression of these molecules in the samples from infected individuals after 7 weeks of infection (Table 1, Figure 4).

#### There Is an Important Upregulation of Proteins Associated With RNA Processing, Translation and Vesicle Transport

The general Reactome pathway of RNA metabolism was significantly enriched after our analysis, including 18.5% of the mapped proteins with most of them upregulated (79.5%). Included in this category, we found the upregulation of nucleolar RNA helicase 2 (Ddx21; Fold-change: +1.31), Nucleolar protein 58 (Nop58l; Fold-change: +1.44), Exportin-1 (Xpo1; Fold-change: +1.98), and several ones related to the specific pathway of RNA Splicing. This last category was represented by various small nuclear ribonucleoproteins (Snrpb2, Snrpn, Snrpa1, U2af2, Hnrnpul1, Snrnp200; Fold-change: +1.75, +1.48, +1.38, +1.31, +1.30, +1.30, respectively), pre-mRNA processing factor 8 (Prpf8; Fold-change: +2.25), poly-U binding factor 60 (Puf60; Fold-change: +1.93), Pleiotropic regulator 1 (Plrg1; Fold-change: +1.73) and the polypyrimidine tract binding protein 1 (Ptbp1; Fold-change: +1.48). Among the downregulated proteins, we found the heterogeneous ribonucleoprotein A0 (Hnrnpa0; Fold-change: −1.91), protein phosphatase 2 (Ppp2ca; Fold-change: −1.41), one RNA binding motif protein (Rbmx; Fold-change: −1.33), and a non-histone chromosome protein (Nhp2l1; Fold-change: −1.28) (Table 1; Figure 3).

In terms of Translation, several ribosomal proteins were found upregulated (Rps10, Fau, Rps17, Rps7, Rpl13a, Rps3; Fold-change: +1.75, +1.53, +1.48, +1.31, +1.24, +1.23, respectively) along with translation factors (Eif2s2, Eif3a, Eif3l, Eif2s1, Eif3f, Eif4a1, Eef2; Fold-change: +2.61, +2.53, +1.67, +1.53, +1.46, +1.30, +1.28, respectively). The ribosomal protein S16 was found downregulated (Rps16l; Fold-change: −1.32) (Table 1; Figure 3).

In this context, several proteins were found upregulated within the pathway of Vesicle-mediated transport. Among them, we observed four RAS-related proteins (Rab10, Rab5c, Rab7, Rab11b; Fold-change: +2.38, +1.55, +1.41, +1.38, respectively), four coatomer complex subunits (Copz1, Arcn1, Copb2, Copa; Fold-change: +3.13, +1.88, +1.62, +1.60, respectively), sorting nexins (Snx5, Snx2; Fold-change: +2.32, +1.36, respectively), charged multivesicular body protein 4B (Chmp4b; Fold-change: +2.10), spectrin beta (Sptb; Fold-change: +1.72) and a mannose-6-phosphate receptor (M6pr: Fold-change: +1.47) (Table 1; Figure 3).

#### Many Proteins Related to Cell Cycle and DNA Replication Are Found Upregulated

The Cell Cycle major pathway was significantly enriched by our set of differentially expressed proteins. Twelve percent of the mapped proteins were included within this category, with 76.9% of them upregulated. Among the most prominent, we found the DNA replication licensing factor MCM6 (Mcm6; Fold-change: +4.80), an actin dependent regulator of chromatin (Smarca5; Fold-change: +2.15), two DNA helicase components (Mcm5, Mcm7; Fold-change: +1.74, +2.10, respectively), proliferating cell nuclear antigen (Pcna; Fold-change: +2.05), the nucleoporin 93 (Nup93; Fold-change: +1.83), lamin A (Lmna; Fold-change: +1.70), the mitotic checkpoint protein BUB3 (Bub3; Fold-change: +1.60) and the histone binding protein 7 (Rbbp7; Fold-change: + 1.31). In contrast, the protein 1A for structural maintenance of chromosomes was found downregulated (Smc1a; Fold-change: −1.79) (Table 1; Figure 3).

#### The Pathways Associated With Catabolic Metabolism Were Mostly Downregulated

Pathways related to the TCA cycle, respiratory transport and amino acid metabolism were predominantly enriched by downregulated proteins. A set of seven dehydrogenase subunits were found in this condition (Pdha1, Ldhb, Sdha, Ndufs4, Idh2, Pdhb, Glud1; Fold-change: −2.36, −2.29, −1.68, −1.32, −1.32, −1.30, −1.31, respectively), as well as Acetyl-CoA acetyltransferase 1 (Acat1; Fold-change: −1.34), creatine kinase (Ckb, Fold-change: −1.33) and a peptidase subunit of the proteasome (Psmb8; Fold-change: −1.27), also enriching pathways of antigen processing and presentation. Conversely, two ubiquinone proteins (Ndufb4, Ndufs1; Fold-change: +2.22, +1.85, respectively), an ATP synthase subunit (Atp5d; Fold-change: +1.45) and adenosine deaminase (Ada; Fold-change: + 1.47) were found upregulated (Table 1, Figure 3).

### Proteins With Exclusive Identifications by Experimental Condition

Although our analyses were mainly focused on proteins that were differentially expressed between Infected and Control individuals, there is an important set of proteins with unique identifications in the two conditions. When comparing the protein sets of identified proteins, it is observed that 289 were exclusively identified in samples from Infected individuals while 263 were found only in the Control group (Supplementary Figure 4). Among the exclusive proteins in the infected condition, we found a set of splicing factors subunits, several proteins related to DNA metabolism and a few proteins representatives of alternative pathways of catabolic metabolism such as arachidonate lipoxygenase. Some of the uniquely identified proteins in the control condition were those associated with mitochondrial metabolism such as cytochrome subunits and NADH dehydrogenase (Supplementary Table 3).

### The Immunophenotyping of Splenocytes Reveals a Change in Cell Proportions and MHC II Presentation by Macrophages

**Figure 5:**
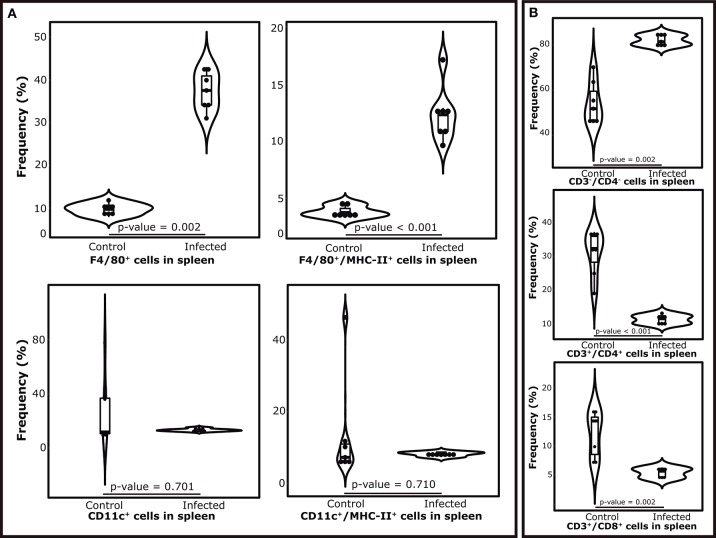
Variations in the cell populations in the spleen after 7 weeks of *S. mansoni* infection in Balb/c mice. (Top section, from left to right) **(A)** Violin plots showing the mean proportions of antigen presenting cells by experimental condition. Upper panel shows the proportions of macrophages (F4/80^+^ cells) and MHC-II-presenting macrophages (F4/80^+^/MHC-II^+^ cells) as a fraction of the whole population of spleen cells. Bottom panel shows the proportion of dendritic cells (CD11c^+^ cells) and MHC-II-presenting dendritic cells (CD11c^+^/MHC-II^+^ cells) as a fraction of the whole population of spleen cells **(B)** Violin plots showing the mean proportions of the subpopulations of T lymphocytes as a fraction of the whole population of spleen cells.

The composition of immune cells in the spleen showed important variations after the establishment of splenomegaly in terms of lymphocyte populations. The mean percentages of both T CD3^+^CD4^+^ and CD3^+^CD8^+^ lymphocytes were decreased in infected individuals (8.7 and 2.85%, respectively). In the same context, CD3^−^ spleen cells (not T-lymphocytes) were found increased during splenomegaly, representing more than 85% of the cell population (Figure 5A). In terms of APCs, we found an increased frequency of macrophages (F4/80^+^ cells) in infected individuals, which was also accompanied by a higher proportion of MHC-II-presenting macrophages (F4/80^+^/MHC-II^+^ cells) in this condition. Finally, no significant differences were found in the proportions of CD11c^+^ dendritic cells during splenomegaly, along with no differences in the numbers of CD11c^+^/MHC II^+^ cells and the expression levels of MHC-II receptors in the latter population (Figure 5B).

## Discussion

Here we aimed to provide the first large-scale quantitative examination of the spleen proteome during murine schistosomiasis caused by *S. mansoni* infection. Using highly sensitive proteomic tools for identification and stringent statistical criteria for label-free quantification, we were able to describe minor variations in the abundance of an important set of proteins in splenocytes. The findings should have implications in the development of the host response during the helminthic infection and the complex dynamic associated with the immune pathology that could potentially lead to establishment of the chronic disease. After 5 weeks of infection, sexual maturation of the parasites is fully accomplished and female worms have started oviposition at the intestinal mesenteric veins (29, 30). Under this condition, some eggs are transported through vein circulation and reach the liver, where they promote an exaggerated immune response in the tissue with the formation of granuloma and the installation of a hepatic fibrosis (8), resulting in the establishment of a hepatosplenomegaly in the murine model (21). In this study we have chosen to investigate the spleen proteome, at the peak of acute phase of inflammation, to add understanding into how the organ responds to the buildup of parasitic antigens in the circulation mostly represented by egg secretions and regurgitation contents of adult parasites.

In this context, we first set up an experimental scheme to distinguish the establishment of splenomegaly in the Balb/c strain. After 7 weeks of infection, spleen histology revealed a marked rearrangement in tissue morphology. Evaluation of the spleen-to-body ratio in infected individuals, compared to control non-infected animals also demonstrated a > 4-fold increase in this parameter. This, along with a high egg count in the feces of infected individuals, allowed us to corroborate the presence of hepatosplenic schistosomiasis in our infection model.

High throughput proteomic sequencing approaches provide a manner to make inferences on the variation of protein abundance under distinct biological conditions. The “bottom-up” label-free shotgun analysis initiates by the proteolysis of complex protein extracts for the production of peptides that are then separated through high-resolution chromatography. Coupled to highly sensitive mass analyzers, it is possible to obtain peptide spectral data that are interrogated for identification and quantification of proteins within a sample (31). This method offers an unbiased approach for the discovery of systematic changes in protein expression and molecular signatures in tissues and cells during particular experimental conditions can be revealed (32). In contrast to classic targeted antibody-based approaches, the study of the immune system through large scale proteomics is potentially useful for a comprehension of the highly interconnected cellular networks associated with the response to infection or disease (33).

In this study, Patternlab for proteomics (23) was employed as the software platform for the identification and label-free quantification of proteins from spleen cells. The compositional analyses showed the complexity of the protein extracts, with a dynamic range of abundance varying over four orders of magnitude. Almost a half of the identified proteins were those of low abundance, representing only 10% of the total mass of the extract. Nevertheless, the use of a stringent criteria allowed the identification of differentially expressed proteins all along the encompassed dynamic range. We then used a volcano plot in order to visualize the 325 proteins that showed a differential abundance of at least 20% (Fold-change ±1.2) between the two conditions. Most of them (242, > 70%) were upregulated in infected individuals and 83 were downregulated. The L-Stringency criteria pinpointed 150 proteins considered to be differentially expressed but presented low spectral counts, artificially decreasing the *p*-value of the comparison of the particular protein between conditions, and possibly leading to false positives (24).

The spleen, as the largest lymphoid organ in the body, contains a complex tissue organization that is anatomically connected to the liver via the portal vein system. An essential association has been described between the development of schistosomiasis and other liver diseases with morphologic modifications and clinical manifestations in the spleen. It has been suggested that these splenic abnormalities may play a key role in the progression and prognosis of liver fibrosis (34). Studies in human patients suggest the spleen as a key site for T-cell activation, with an important exchange of cells with the liver during *S. mansoni* infection (35, 36). However, the precise mechanisms governing the interphase of the immune modulation in the spleen and the hepatosplenic pathology are still poorly understood.

In our study, both the Reactome and KEGG databases were used for the functional characterization of the set of differentially expressed proteins in spleen cells, with emphasis in the quantitative information in cases of positive and negative regulation. Using this approach, we were able to evaluate the simultaneous variation in abundance of proteins enriching specific pathways in the context of splenomegaly at the acute phase of inflammation. It was observed that most upregulated proteins were categorized in pathways related to the immune system, RNA processing, translation, vesicle transport and cell cycle. Specifically, the pathways of innate immunity and neutrophil degranulation showed a positive regulation of eosinophil-associated ribonucleases, eosinophil cationic proteins, eosinophil peroxidase and bone marrow proteoglycan 2. All of these proteins are featured within the eosinophil granules and have been associated with the response to allergens, asthma and helminth infections (37, 38). In relation to *S. mansoni*, the eosinophil granule proteins have been described as highly cytotoxic against schistosomula *in vitro* (39). In accordance, eosinophils are found to be major components of the liver granuloma and, specifically, the eosinophil cationic protein (ECP; Ear2 in the mouse) has been studied as an important factor involved in the defense and prognosis of hepatic fibrosis during *S. mansoni* infection in humans. Highly cytotoxic isoforms of this protein tend to be related with a lower parasitic burden and a reduction of morbidity associated with hepatic fibrosis in endemic areas (40).

When focusing on the comparative analysis of the spleen proteome, it was noticeable the increased contribution of the eosinophil granule proteins in infected individuals. The eosinophil peroxidase (Epx) and the bone marrow proteoglycan 2 (Prg2) happened to be among the one hundred most abundant proteins in this condition. Respectively, Epx has been described as the only protein uniquely expressed in eosinophils, while Prg2 is among the top 10 most abundant proteins in these granulocytes (41). In similar sense, studies on the development of the immune pathology have demonstrated the requirement of particular Th2 cytokines for the consequent differentiation and activation of eosinophils (42, 43). These have been classically associated with an eosinophilia peak in the murine model after 7–8 weeks of primary infection (44). Altogether, the most plausible explanation for the presence of eosinophil proteins in the spleen proteome is due to a high blood eosinophilia, noticing the extensive vascularity of this organ. Although the eosinophils have been described to be implied in processes of antigen presentation and T cell activation (45, 46), our current approach does not allow for further inferences on a possible process of cell recruitment and the presence of an eosinophil infiltrate in the spleen. Alternatively, we cannot rule out the possibility that these proteins are also expressed by other spleen resident cells. Inspection of public databases, showed the expression of these proteins in macrophages and dendritic cells after whole-genome expression analyses based on RNA-seq and microarrays (47, 48).

In terms of adaptive immunity, the categorization analyses showed enrichment of pathways related to both MHC class I and II of antigen presentation. This label-free shotgun approach confidently measured the upregulation of several H-2 class I histocompatibility antigen alpha chains, chaperone proteins involved in folding, assembly and peptide loading and one activator subunit of the immunoproteasome. Along with these observations, it was found an important upregulation of class II histocompatibility antigens and a significant set of proteins associated with the activation, degradation, processing and transit of external proteins. The development of the immune pathology during the *S. mansoni* infection is characterized by an intense polarization from a Th1 to a Th2 immune response which is associated with the complete maturation of worms and the start of the oviposition (29). In this context, our findings revealed increased levels for MHC class II molecules compared to MHC class I, which is consistent with the priming of CD4^+^ T cells and the development of T helper-dependent immune responses (49).

This complex immune dynamic after a helminthic infection has been related to mechanisms of host-parasite co-evolution, that allows the parasite to impair a totally effective immune response from the host against its presence. Both adult worms and eggs have an important antigenic contribution, and several excretory and secretory molecules from helminth parasites have been associated with particular modulatory functions and mechanisms of immune evasion (50), such as the proteinase inhibitors serpin and cystatin (51). In accordance, we found the downregulation of key proteins for the processes of antigen processing and presentation and neutrophil recruitment and degranulation. Two serine proteases (neutrophil elastase-Elane and proteinase-3-Prtn3) and one cysteine protease (Cathepsin B-Ctsb) were found at decreased levels. A recent study on the *S. mansoni* serine protease inhibitor *Sm*KI-1, points out the implication of this molecule on the decreased activity of neutrophil elastase (Elane) and the consequent impairment of neutrophil activity in various models of inflammation (52). Although their role in MHC class II antigen presentation is still discussed, Elane and Prtn3 are expressed in the azurophilic granules of neutrophils and are thought to be involved in the clearance of internalized pathogens and the activation of cell receptors (53). Continuing, Ctsb is deemed relevant in the lysosomal processing of external antigens for their further presentation by antigen presenting cells. Its inhibition has been recently associated with the activation of APC-mediated anti-inflammatory immunity in hepatic pathology (54). Here we also reported the negative regulation of two subunits of the proteasome: the β8 subunit of the immunoproteasome (Psmb8) and the canonical β4 from the 20S core complex (Psmb4) suggesting a possible regulatory process related to proteasome-mediated protein degradation. Nonetheless, further experimentation and validation on these latter observations are recommended, since the quantification of Psmb8 was marked by our L-stringency criteria due to its low spectral abundance.

Along with a conspicuous enrichment of pathways related to antigen presentation, we observed upregulation of proteins associated with transcription, translation and vesicle transport. These are also in conjunction with increased expression of several proteins related to pathways of cell cycle, metabolism of DNA and replication such as DNA helicase, replication licensing factors and adenosine deaminase. Among other proteins of RNA synthesis and processing, the positive regulation of a handful of small nuclear ribonuleoproteins and other proteins involved in alternative RNA splicing were detected. This is consistent with an active immune response, which requires the diversity and plasticity of effector cells for the production of antibodies, receptors and adhesion molecules (55) to sustain the demand for T cell differentiation and proliferation, leading to the development of splenomegaly in this condition. In the same context, we found a significant downregulation of proteins within pathways associated with catabolic metabolism, suggesting a metabolic reprogramming that has been related to the activation and differentiation of immune cells (56, 57).

A notable caveat of a shotgun investigation applied to a whole organ or tissue refers to the difficulty in assigning a particular protein expression to a given cell population. In this context, proteomic data combined with RNA-seq analyses directed to a specific cell type can potentially provide relevant clues to segregate the observed differential expression of proteins associated with a particular condition. Another approach is the use of flow cytometry, provided specific antibody cell markers are available. Here, we employed immunophenotyping of spleen cells in attempt to initiate the establishment of an association between the proteomic results with the concurrent variations in the population of immune cells, at the peak of the acute phase during schistosomiasis in the murine model. At first, an important decrease in the contribution of T lymphocytes to the total population of splenocytes is perceived. Both CD4^+^ and CD8^+^ T cells are found in minimal proportions during splenomegaly, while non-T CD4^−^/CD8^−^ cells take the leading role in abundance. It was showed and increased contribution of macrophages to the cell population, whilst other antigen presenting cells (CD11c^+^) remained unchanged. Taking into account the predominant cells in the spleen constitution, the decreased number of CD4^+^ and CD8^+^ T lymphocytes could suggest an increased proportion of B lymphocytes which agrees with the expansion of the lymphatic follicles observed in the spleen. This is consistent with the activation of the previously described processes of cell differentiation and protein synthesis and could be associated with the peaks in antibody titers in mice serum after 7 weeks of infection (58). In the context of antigen presentation, we found that macrophages seem to have an important role as MHC II-presenting cells, pointing out that the increased expression of MHC II molecules, measured by mass spectrometry, could be associated with the increased proportion of these cells in the spleen.

This study represents the first discovery approach based on a LC-MS/MS shotgun analysis of the spleen proteome during the infection by *S. mansoni*. The findings bring up novel hypotheses on the influence of parasitic antigens for the development of the immune pathology associated with schistosomiasis. In this context, a set of modifications in the molecular dynamics and cellular composition of the spleen, at the peak of the acute phase of infection, mostly associated with immunomodulatory processes were appointed. We are reporting the potential effect of the infection on several proteins within pathways of antigen processing and presentation, RNA processing, cell cycle and catabolic metabolism. Noticing the currently poor understanding of the implications of the spleen in the development of schistosomiasis, we believe that this study represents a starting point for determining the molecular signatures produced by the host in response to the infection. Future research could be pointed out to the expansion of this study to other stages of the infection (i.e., pre-patent and chronic), accounting for the variable antigenic contribution from the different stages of the parasite, as a way of improving the representation of the complex host-parasite interplay during the course of schistosomiasis.

## Data Availability Statement

The mass spectrometry proteomics data, including pre-processed results and R scripts for data analysis, have been deposited to the ProteomeXchange Consortium via the PRIDE (59) partner repository with the dataset identifier PXD011153.

## Author Contributions

MC-C and WC-B designed the study, analyzed and interpreted data and wrote the manuscript. MC-C, RO, JC, and GG conducted experiments, analyzed and contributed to data interpretation. BM, NP, and ROA-S conducted instrumental analysis, offered technical expertise and contributed to interpretation of data. CC and LA provided reagents and contributed to interpretation of data. All authors reviewed the manuscript.

### Conflict of Interest Statement

The authors declare that the research was conducted in the absence of any commercial or financial relationships that could be construed as a potential conflict of interest.

## Supplementary Material

Supplementary Figure 1 Experimental design flowchart.

Supplementary Figure 2 Correlation panel demonstrating the reproducibility of the protein quantifications between biological replicates. “Corr” values represent the Pearson's r-squared values of correlation.

Supplementary Figure 3 Strategies of analysis used to identify specific subpopulations of spleen cells as depicted by the pseudocolor bi-dimensional fluorescence graphs. **(A)** Forward scatter (FSC) vs. Side scatter (SSC) plot as a representation of the whole population of spleen cells. **(B)** B-lymphocyte population as identified by the set of CD19^+^/SSC events. **(C)** The T-lymphocytes as identified by the set of double positive events for CD3^+^/CD4^+^ or CD3^+^/CD8^+^ cells (upper-right quadrant). **(D)** Gating strategy for the identification of CD11c^+^-SSC events followed by MHC-II-presenting dendritic cells. **(E)** Gating strategy for the identification of F4/80^+^SSC events followed by MHC-II-presenting macrophages.

Supplementary Figure 4 Venn diagram for the set of proteins identified in both non-infected (Control) and Infected mice, after 7 weeks of *S. mansoni* infection.

Supplementary Table 1 Summary statistics for flow cytometry data. Number of events and frequencies of spleen cells subpopulations.

Supplementary Table 2 Quantitative data on the protein expression levels in spleen cells after 7 weeks of *S. mansoni* infection.

Supplementary Table 3 Set of uniquely identified proteins in spleen cells after 7 weeks of *S. mansoni* infection and in Control individuals.
