# Supplementary figures and images for: The Schistosomiasis SpleenOME: Unveiling the Proteomic Landscape of Splenomegaly Using Label-Free Mass Spectrometry
Source: Front Immunol. 2019 Jan 22;9:3137. doi: 10.3389/fimmu.2018.03137 (PMC6352917; doi:10.3389/fimmu.2018.03137)

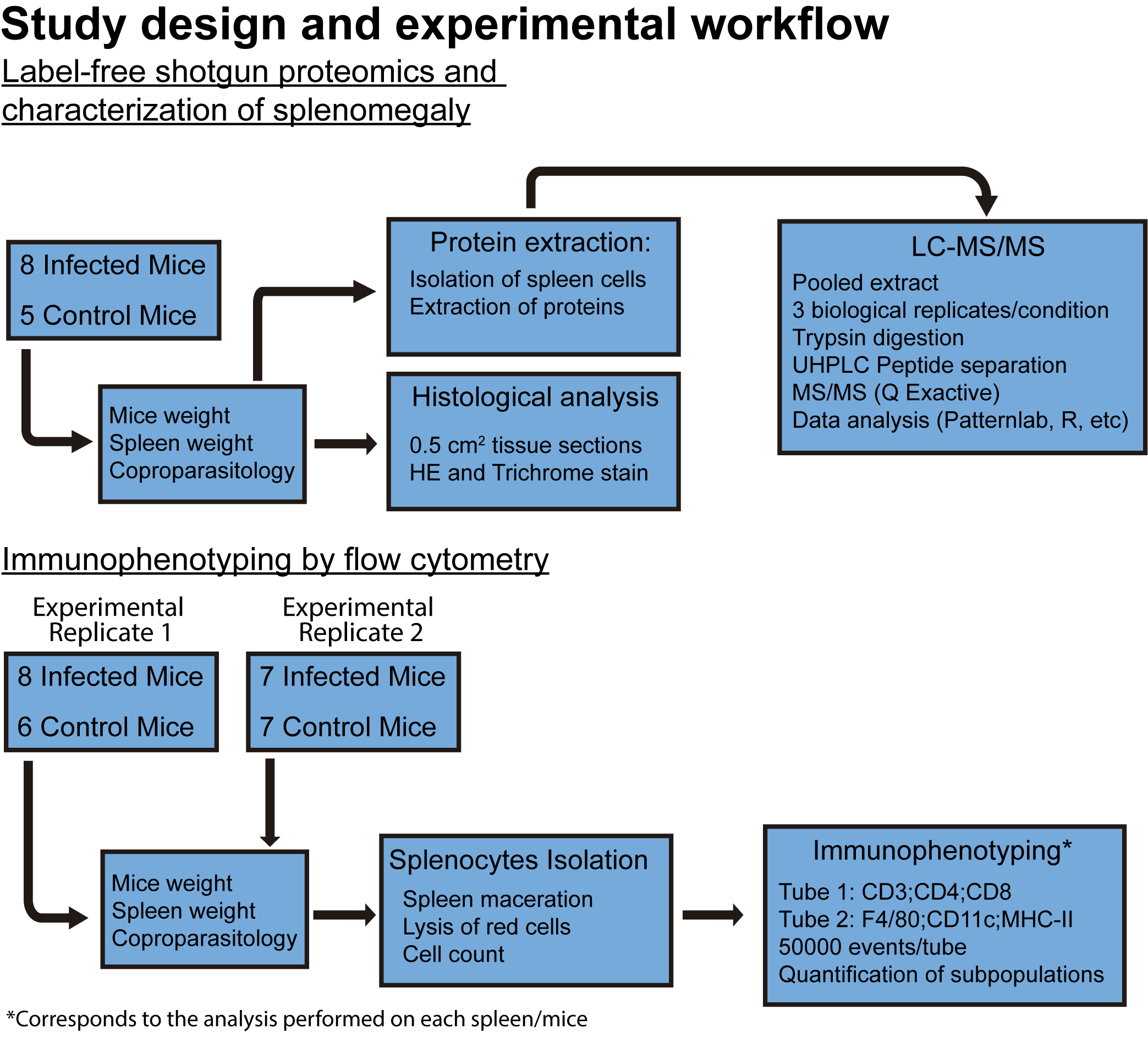

Supplement: Supplementary Figure 1 — Experimental design flowchart. [file Image_1.tif]

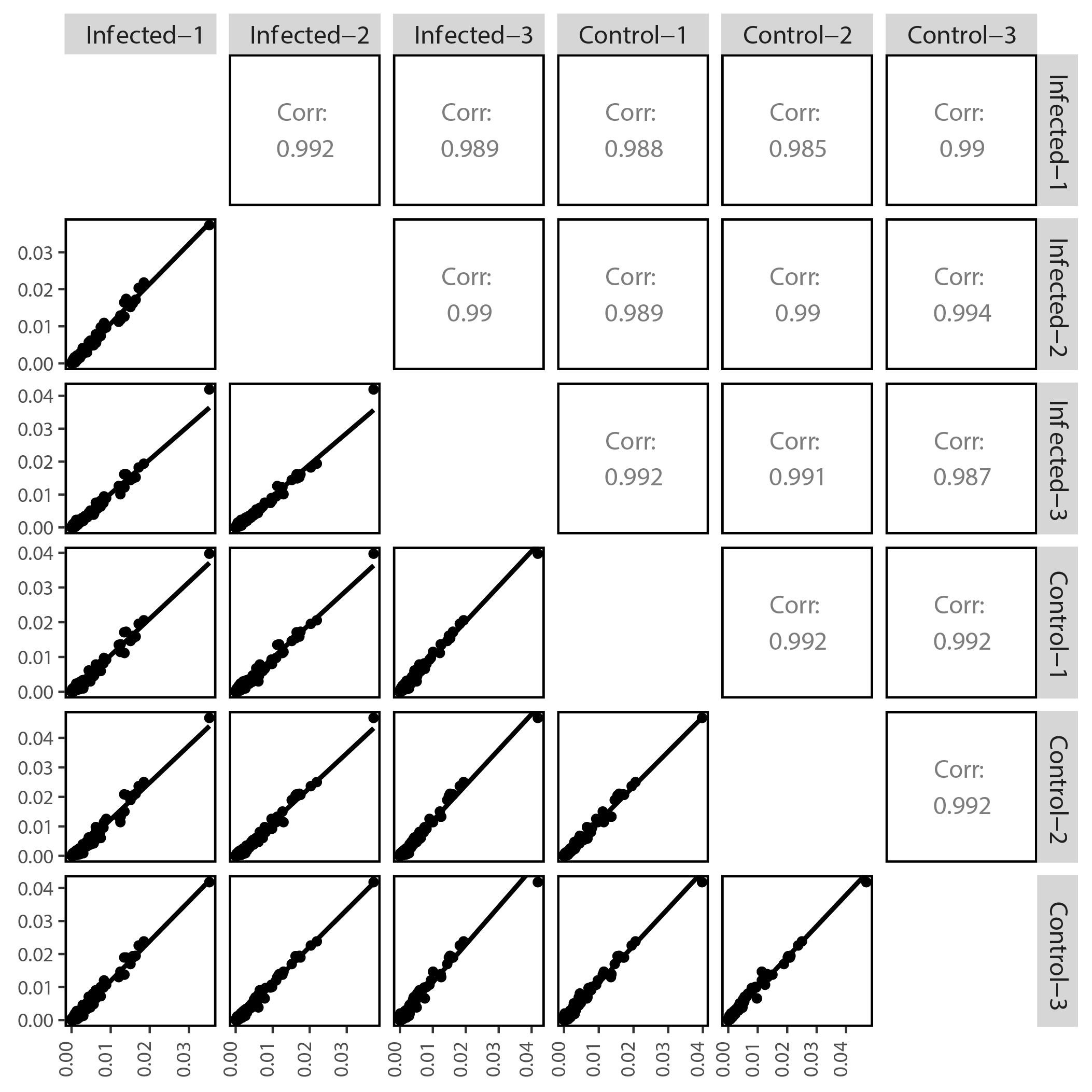

Supplement: Supplementary Figure 2 — Correlation panel demonstrating the reproducibility of the protein quantifications between biological replicates. “Corr” values represent the Pearson's r-squared values of correlation. [file Image_2.tif]

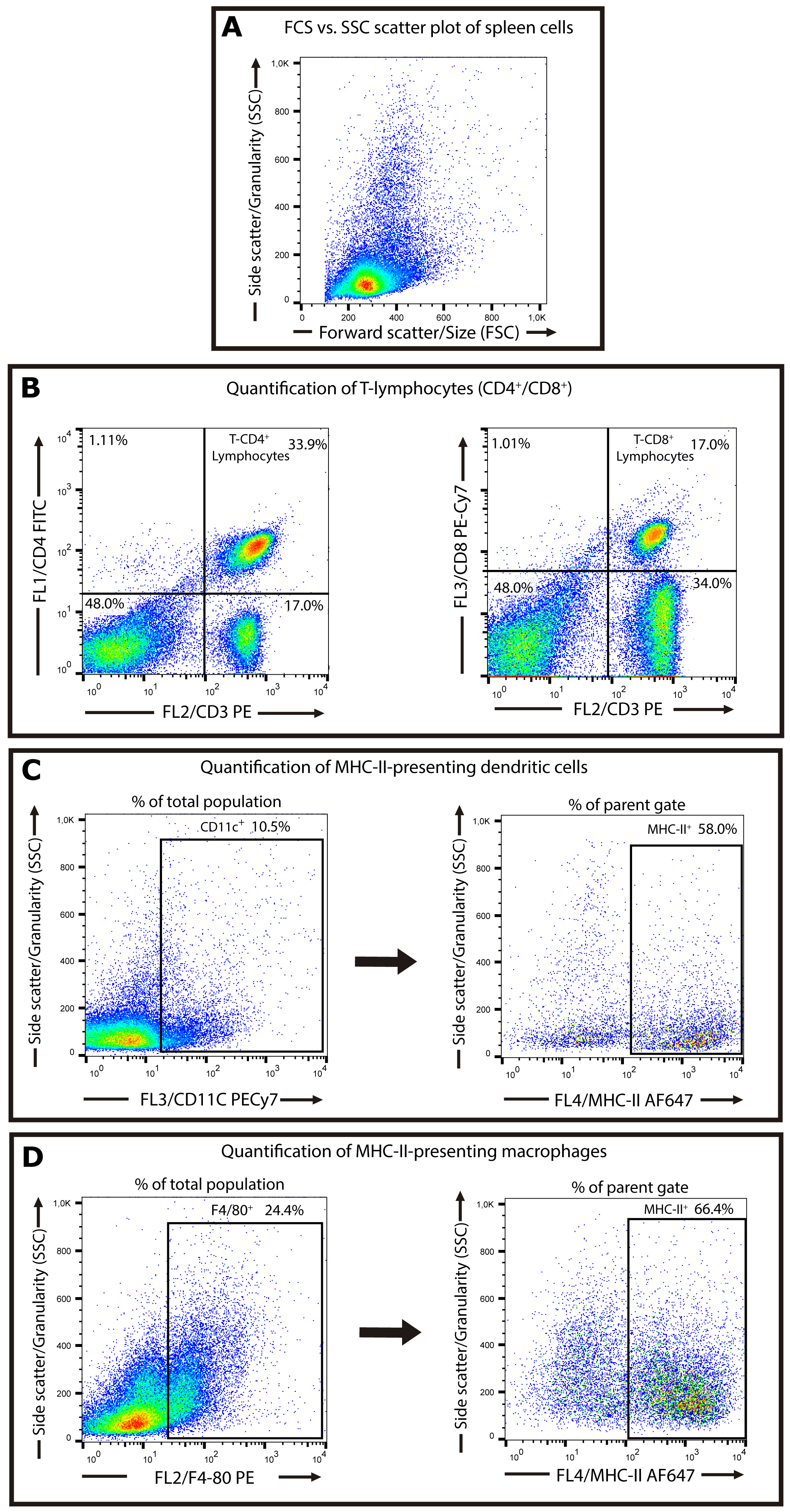

Supplement: Supplementary Figure 3 — Strategies of analysis used to identify specific subpopulations of spleen cells as depicted by the pseudocolor bi-dimensional fluorescence graphs. (A) Forward scatter (FSC) vs. Side scatter (SSC) plot as a representation of the whole population of spleen cells. (B) B-lymphocyte population as identified by the set of CD19+/SSC events. (C) The T-lymphocytes as identified by the set of double positive events for CD3+/CD4+ or CD3+/CD8+ cells (upper-right quadrant). (D) Gating strategy for the identification of CD11c+-SSC events followed by MHC-II-presenting dendritic cells. (E) Gating strategy for the identification of F4/80+SSC events followed by MHC-II-presenting macrophages. [file Image_3.tif]

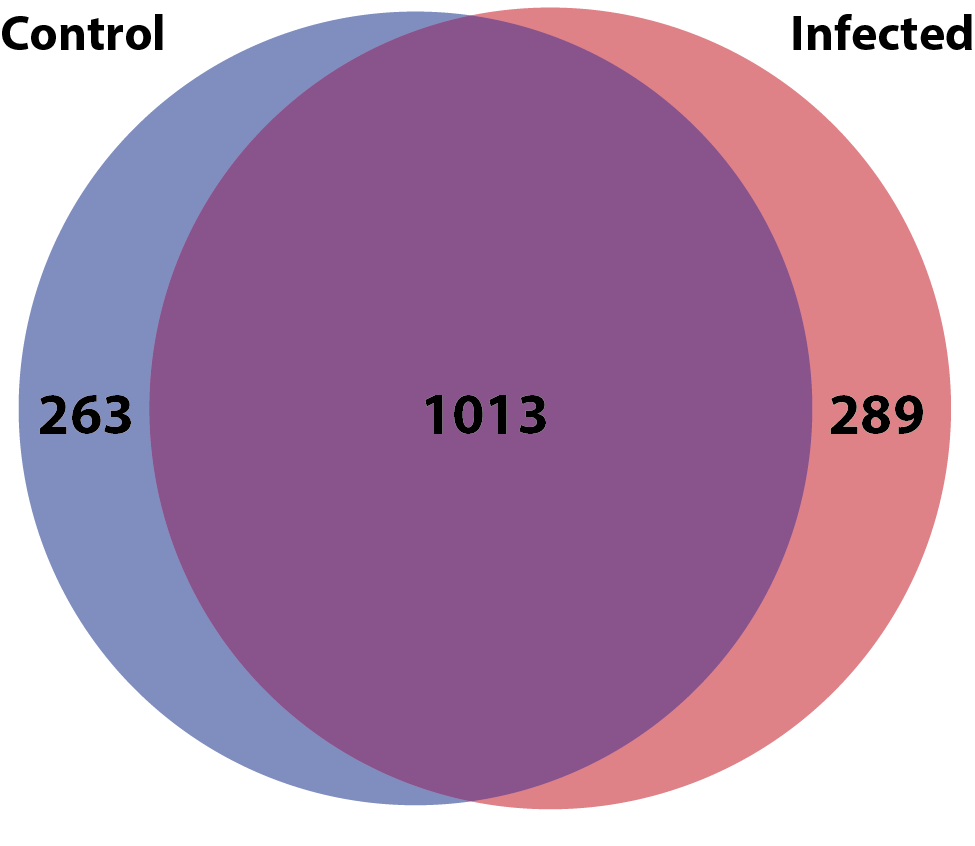

Supplement: Supplementary Figure 4 — Venn diagram for the set of proteins identified in both non-infected (Control) and Infected mice, after 7 weeks of S. mansoni infection. [file Image_4.tif]
